# How Much Do Young Italians Know About COVID-19 and What Are Their Attitudes Toward SARS-CoV-2? Results of a Cross-Sectional Study

**DOI:** 10.1017/dmp.2020.205

**Published:** 2020-06-24

**Authors:** Giuseppe La Torre, Lorenza Lia, Barbara Dorelli, Mattia Marte, Marta Chiappetta, Augusto Faticoni, Lorenzo Lucaccini Paoli, Daniele Grassucci, Marcello Gelardini, Carla Ardizzone

**Affiliations:** Department of Public Health and Infectious Diseases, Sapienza University of Rome, Italy; Skuola Network Srl

**Keywords:** attitudes, COVID-19, knowledge, SARS-CoV-2, young Italians

## Abstract

**Objectives:**

At the end of 2019, an outbreak of novel coronavirus pneumonia, called severe acute respiratory syndrome coronavirus 1 (SARS-CoV-2), was first identified in Wuhan, Hubei Province, China. It subsequently spread throughout China and elsewhere, becoming a global health emergency. In February 2020, the World Health Organization (WHO) designated the disease coronavirus disease 2019 (COVID-19). The objective of this study was to investigate the degree of knowledge of young Italians about COVID-19 and their current attitudes toward the SARS-CoV-2 and to determine if there were prejudices emerging toward Chinese.

**Methods:**

An online survey was conducted on February 3, 4, 5, 2020, with the collaboration of Italian website “Skuola.net”. Young people had the opportunity to participate by answering an ad hoc questionnaire created to investigate knowledge and attitudes about the new coronavirus, using a link published on the homepage.

**Results:**

A total of 5234 responses were received, of which 3262 were females and 1972 were males. Most of the participants showed generally moderate knowledge about COVID-19. Male students, middle school students, and those who do not attend school, should increase awareness of the disease; less than half of responders say that their attitudes toward the Chinese population has worsened in the last period.

**Conclusions:**

Global awareness of this emerging infection should be increased, due to its virulence, the significant risk of mortality, and the ability of the virus to spread very quickly within the community.

Coronaviruses (CoVs), important human and animal pathogens, are a family of RNA viruses that typically cause mild respiratory, enteric, hepatic, and neurologic disease in humans.^1,2^ Six coronavirus species are known to cause human disease: among these, 4 species, including hCoV-229E, OC43, NL63, and HKU1, are prevalent and typically cause mild respiratory diseases,^3^ while 2 novel fatal coronaviruses emerge periodically in different areas: severe acute respiratory syndrome coronavirus (SARS-CoV) and Middle East respiratory syndrome coronavirus (MERS-CoV).

At the end of 2019, an outbreak of novel coronavirus pneumonia, called SARS-CoV-2, was first identified in Wuhan, Hubei Province, China.^4,5^ It is supposed that the new virus originated from an animal-to-human spillover event linked to seafood and live-animal markets. It subsequently spread throughout China and elsewhere, becoming a global health emergency. In February 2020, the World Health Organization (WHO) designated the disease COVID-19, which stands for coronavirus disease 2019.^6^

Coronavirus infection in humans causes mild to moderate respiratory diseases, such as colds, that last for a short period of time. Symptoms may include runny nose, cough, inflamed throat, fever, headache, gastrointestinal disorders, and general malaise. Human coronaviruses can cause diseases of the lower respiratory tract, such as pneumonia or bronchitis. This occurs mainly in people suffering from pre-existing chronic diseases of the cardio-vascular and/or respiratory system, and individuals with a weakened immune system, in infants and the elderly.

The transmission of the virus takes place from 1 infected person to another through: saliva, coughing, and sneezing, with direct personal contact and touching with contaminated hands (not yet washed) mouth, nose, or eyes. The contagion can occur through fecal contamination.^7^

There are many gaps in the knowledge of the epidemiology, prevalence, and clinical manifestation of infection. As the World Health Organization (WHO) has pointed out, the transmission of adequate information to the public about the virus, how contagion happens and appropriate prevention measures are essential to ensure adequate disease control.^8^

The new COVID-19 virus is still little known and this characteristic leads on the 1 hand to look for as much information as possible on the subject, on the other hand, to rely on sources that often report incorrect or unfounded news.

However, the need to know and inform is associated with a loss of confidence in institutions, science, and medicine, and the interests of pharmaceutical companies are feared. In the age of social networks, people are subject to an informational deluge, and new forms of media epidemics are developing that quickly transmit habits and behaviors, even wrong and unfounded news.^9^

The aim of this study was to investigate the degree of information on new coronavirus among young Italians and to understand if young people in Italy had developed prejudices against the Chinese because of the coronavirus.

## METHODS

### Study Design and Participants

This study was a cross-sectional study. An online survey was conducted on February 2020 with the collaboration of “Skuola.net”, an Italian website for information and insights for secondary school, high school, and university students.

The students and also people who do not attend any school or university but simply viewed the website, had the opportunity, during 3 d, to participate in the survey by answering the questionnaire through a link published on the homepage (www.skuola.net). No limits of age were applied to the participants. This study was carried out according to the STROBE statement.^10^

### Data Collection

The questionnaire was created to investigate the knowledge and attitudes of Italian students about the new coronavirus (SARS-CoV-2), on the basis of previous published studies.^8,11-13^ It was developed in Italian language and composed by 16 multiple choice questions: 7 concerning knowledge, 5 concerning attitudes and, 4 about socio-demographic data, such as gender, age, school, and geographic area. The participants were assured about the anonymity of their responses. The data from survey responses were collected in an Excel file for statistical analysis. A pilot study involving 93 students revealed a Spearman correlation coefficient for test-retest reliability of 0.908 (*P* < 0.001) and Chronbach alpha of 0.701.

### Statistical Analysis

All analyses were performed using SPSS for Windows (Statistical Package for the Social Sciences, Version 25; SPSS, Inc., Chicago, IL). A descriptive analysis of the categorical variables was conducted using absolute frequencies and percentages. The associations among sex, school, geographic area, and attitudes and knowledge were evaluated. The differences between groups with respect to the categorical variables were analyzed using the Chi-square test. A score was created using the correct answers to the 7 knowledge questions evaluated with 1-point, with a range of value from 0 (no knowledge) to 7 (maximum knowledge): we considered for good knowledge a cutoff of correct answers of 75% (5/7).

A multivariate linear regression analysis was performed using a forward stepwise selection, considering the score of knowledge as dependent variables and socio-demographic factors as independent variables. The goodness of fit for the model was assessed with R2.

Moreover, 5 logistic regression models were computed, estimating odds ratio (OR) with 95% confidence intervals (95% CIs): the dependent variable in the models was each question about attitudes, and the independent variables were age, sex, school, geographic area and knowledge score. Significance threshold was set at *P* < 0.05 for all analyses.

## RESULTS

### Descriptive Analysis of the Sample

A total of 5374 people took part in the questionnaire and 5234 complete answers were received, of which 3262 (60.7%) were females and 1972 (36.7%) were males. The frequencies of the socio-demographic characteristics (age, attended school, regional macroarea) of the sample are shown in Table 1.

**TABLE 1:** Selected Demographics Characteristics of the Respondents

Variables	*N* (5234)	(%)
Gender		
Female	3262	(60.7)
Male	1972	(36.7)
Age, years		
11-13	503	(9.4)
14-16	1510	(28.1)
17-19	1440	(26.8)
20-22	507	(9.4)
23-25	274	(5.1)
25-30	1140	(21.2)
Attended school		
Middle school	652	(12.1)
High school (first 2)	1048	(19.5)
High school (last 3 y)	1662	(30.9)
University	957	(17.8)
Not attending school/university	946	(17.6)
Macroarea of Italy		
North	2413	(44.9)
Center	1205	(22.4)
South	1637	(30.5)

### Univariate Analysis: Knowledge Questions

Questions 1 to 7 concerned knowledge about new coronavirus. To the question “Can coronavirus infection pass from man to man through cough-borne droplets?” especially females answered correctly (82.2%), students attending high school (83.4%), and people from North of Italy (82%). Similar results came from question 2 (“Are fever and cough among the signs and symptoms of coronavirus infection?”, from question 4 (“How long can infection develop after exposure to coronavirus?”), from question 5 (“Can patients with coronavirus infection be cured?”), and from question 7 (“Is there currently a vaccine against coronavirus?”) in which most of the females correctly answered, most of the university students, and most of the people of northern Italy. Table 2 and the additional file Supplemental Table 2A show the results in detail.

**TABLE 2:** Frequency of Answers

Questions	*N* (5234)	(%)
*Q#1: Can coronavirus infection pass from person to person through cough-borne droplets?*		
Yes	4291	(79.8)
No	427	(7.9)
I do not know	656	(12.2)
*Q#2 Are fever and cough among the signs and symptoms of coronavirus infection?*		
Yes	4565	(84.9)
No	271	(5.0)
I do not know	538	(10.0)
*Q#3: Is diarrhea among the signs and symptoms of coronavirus infection?*		
Yes	576	(10.7)
No	3279	(61.0)
I do not know	1519	(28.3)
*Q#4: How long can infection develop after exposure to coronavirus?*		
1day	467	(8.7)
2 days	529	(9.8)
5days	875	(16.3)
10-15 days	3371	(63.7)
I do not know	132	(2.5)
*Q#5: Can patients with coronavirus infection be cured?*		
Yes	3206	(59.7)
No	1044	(19.4)
I do not know	1124	(20.9)
*Q#6: Is there currently evidence that pet animals can transmit coronavirus infection?*		
Yes	572	10.6
No	3235	(60.2)
I do not know	1567	(29.2)
*Q#7: Is there currently a vaccine against coronavirus?*		
Yes	346	6.4
No	4303	(80.1)
I do not know	725	(13.5)
*Q#8: If there were a vaccine available, would you get vaccinated?*		
Yes	3890	(72.4)
No	554	(10.3)
I do not know	930	(17.3)
*Q#9: How has your attitude toward Chinese restaurants changed in this period?*		
It is the same	3616	(67.3)
It has improved	190	(3.5)
It is gotten worse	1407	(26.2)
I do not know	161	(3.0)
*Q#10: How has your attitude toward Chinese-run shops changed in this period?*		
It is the same	3754	(69.9)
It has improved	184	(3.4)
It is gotten worse	1278	(23.8)
I do not know	158	(2.9)
*Q#11: How has your attitude toward Chinese tourists changed in this period?*		
It is the same	3106	(57.8)
It has improved	177	(3.3)
It is gotten worse	1936	(36.0)
I do not know	155	(2.9)
*Q#12: If in the class you attend, there were a student of Chinese origin, how would you behave?*		
It is the same	4167	(77.5)
I would avoid contacts	564	(10.5)
I would urge them not to come	276	(5.1)
I would put on the mask	210	(3.9)
I do not know	157	(2.9)

### Univariate Analysis: Attitudes Questions

Questions 8 to 12 concerned attitudes toward the virus and the Chinese population. Table 2 and the additional file Supplemental Table 2B show the results in detail.

About question number 8 (“If there were a vaccine available, would you get vaccinated?”), 1501 males (71.1%), 2389 females (73.2%), 659 who attending university (68.9%), 2267 who attending high school (83.7%), 511 who attending middle school (78.4%), 453 who not attending any school (42.9%), 1804 from north (74.8%), 903 from center (68.2%), and 1183 from south (72.3%) would agree with the idea of getting vaccinated.

About question number 9 (“How has your attitude toward Chinese restaurants changed in this period?”), 560 males (26.5%), 847 females (26.0%), 201 who attending university (21.0%), 769 who attending high school (28.4%), 228 who attending middle school (35.0%), 209 who not attending any school (19.8%), 627 from north (26.0%), 358 from center (27.0%), and 422 from south (25.8%) say that their opinion on Chinese restaurants has worsened in the last period.

About question number 10 (“How has your attitude toward Chinese-run shops changed in this period?”), 532 males (25.2%), 746 females (22.9%), 208 who attending university (21.7%), 672 who attending high school (24.8%), 213 who attending middle school (32.7%), 185 who not attending any school (17.5%), 546 from north (22.6%), 307 from center (23.2%), and 425 from south (26.0%) say that their opinion on Chinese-run shop has worsened in the last period.

About question number 11 (“How has your attitude toward Chinese tourists changed in this period?”), 828 males (39.2%), 1108 females (34.0%), 297 who attending university (31.0%), 1079 who attending high school (39.8%), 298 who attending middle school (45.7%), 262 who not attending any school (24.8%), 887 from north (36.8%), 499 from center (37.7%), and 550 from south (33.6%) say that their attitude on Chinese tourists has worsened in the last period.

About question number 12 (“If in the class you attend, there were a student of Chinese origin, how would you behave?”), 94 males (4.5%), 116 females (3.6%), 28 who attending university (2.9%),95 who attending high school (3.5%), 49 who attending middle school (7.5%), 38 who not attending any school (3.6%), 97 from north (4.0%), 34 from center (2.6%), and 79 from south (4.8%) answer this question that they would put on a mask in the classroom if a student of Chinese origin was present.

### Multivariate Analysis

Linear regression analysis (Table 3) was performed to evaluate the association between the score of knowledge and socio-demographic variables (age, sex, geographic area, school). All variables were directly associated with the score except for the variables “Macroarea-Center” and “Middle school,” which had no significant relationships (*B* = −0.008; *P* = 0.619; *B* = 0.024; *P* = 0.357).

**TABLE 3:** Linear Regression Model of Score of Knowledge

Independent Variables	Score of Knowledge				
	*B*	Stand. error	*Beta stand.*	t	*P (Sign.)*
Age	0.046	0.023	0.052	2.031	**0.042**
Sex^a^					
Female	0.201	0.041	0.066	4.940	**<0.001**
Macroarea^b^					
North	0.237	0.047	0.079	5.068	**<0.001**
Center	−0.027	0.054	−0.008	−0.497	0.619
Attended school^c^					
University	0.716	0.071	0.185	10.056	**<0.001**
High school	0.498	0.088	0.168	5.627	**<0.001**
Middle school	0.110	0.120	0.024	0.921	0.357

a Reference sex: male.

b Reference macroarea: south.

c Reference attended school: not attending.

Logistic regression models are reported in Table 4. Regarding question 8, the variable “Center” had no significant association (OR, 0.879; CI: 0.756-1.022), while “Age” had an indirect association (OR, 0.666; CI: 0.637-0.697). Center was not significantly different from south Italy, while age had an inverse association with the intention of being vaccinated. The same happened in questions 9 and 11 with the geographic variable “Center,” which was associated with a negative attitudes (OR, 0.796; CI: 0.695-0.913; OR, 0.787; CI: 0.693-0.895). Age was also inversely associated with the attitude investigated in question 12 (OR, 0.943; CI: 0.903-0.986), so the older participants tended to have negative behaviors toward a Chinese-born schoolmate.

**TABLE 4:** Logistic Regression Models of Attitudes Questions

	Q#8: If there were a vaccine available. would you get vaccinated?	Q#9: How has your attitude toward Chinese restaurants changed in this period?	Q#10: How has your attitude toward Chinese-run shops changed in this period?	Q#11: How has your attitude toward Chinese tourists changed in this period?	Q#12: If in the class you attend, there were a student of Chinese origin, how would you behave?
Variable	OR (95% CI)	OR (95% CI)	OR (95% CI)	OR (95% CI)	OR (95% CI)
*Gender*	-				
		ref	ref	ref	ref
Male					
		1.183 (1.047 - 1.337)	1.335 (1.179 - 1.512)	1.455 (1.299 - 1.630)	1.506 (1.310-1.731)
Female					
*Age*	0.666 (0.637 - 0.697)	1.098 (1.054 - 1.144)	1.131 (1.085 - 1.179)	1.156 (1.117 - 1.197)	0.943 (0.903-0.986)
*Macroaerea*					
North	-	-	-	-	1.218 (1.057-1.403)
Center	0.879 (0.756 - 1.022)	0.796 (0.695 - 0.913)	1.208 (1.066 - 1.370)	0.787 (0.693 - 0.895)	-
South	ref	ref	ref	ref	ref
*Attended school*					
High school	1.886 (1.592 - 2.234)	1.172 (1.013 - 1.356)	1.241 (1.081 - 1.424)	-	1.986 (1.684-2.341)
University	1.532 (1.280 - 1.832)	1.370 (1.135 - 1.655)	-		1.899 (1.541-2.339)
Not attending	ref	ref	ref		
					ref
*Corona score*	1.383 (1.323 - 1.447)	1.240 (1.191 - 1.291)	1.234 (1.185 - 1.285)	1.120 (1.079 - 1.163)	1.597 (1.525-1.672)

## DISCUSSION

The aim of this study is to investigate the degree of knowledge of Italian students of middle school, high school, and university about the new coronavirus and their current attitudes toward the coronavirus and the Chinese population, especially if they have changed in the last period. The survey was conducted in early February before the outbreak of the coronavirus epidemic in Italy.

The study highlights how, in particular, female students, university students, high school students, and those of Northern Italy, compared with their respective counterparts, have a greater knowledge about the infection of the new coronavirus and underlines, in particular, how the attitudes and behaviors of male students, middle school students, and those of Central-Southern Italy, toward the Chinese population, have worsened in the last period.

Therefore, greater attention should be paid to male students, middle school students, and those who do not attend school, to increase awareness of the disease and to implement the most suitable preventive measures, designed to stem the spread of the infection.

A similar study of coronavirus, causing MERS, showed good knowledge of the clinical manifestations of MERS-CoV and less knowledge of the epidemiological characteristics of the disease; in addition, the Internet was the main source of information about the infection for respondents to the survey.^8^

A cross-sectional study, carried out on health personnel from the southern region of Saudi Arabia, demonstrated limited knowledge of health personnel regarding the microbiological characteristics of the coronavirus causing MERS.^13^

A cross-sectional study, conducted on 384 participants recruited in various parts of the government of Al-Jouf, Saudi Arabia, included in the questionnaire, unlike our study, more socio-demographic data, such as the origin of the participants from urban or rural areas and a greater number of very useful questions to prevent and stem the spread of the infection: questions regarding the knowledge of the main preventive measures, the treatment of the disease and the epidemiological characteristics of the infection. In addition, they included a question about the main source of information, which appears to be the Ministry of Health, followed, in order of frequency, by the social networks.^14^

Another study that involved medical students highlighted how these students had a good knowledge of the clinical manifestations of the MERS but a poor knowledge of the rate of mortality of the infection.^15^

Our study is the first that evaluates the knowledge of Italian students regarding COVID-19 infection, highlighting a good global knowledge; in addition, only students who visited the “Skuola.net” site were able to participate in the survey by filling in a questionnaire on a link published on the website homepage. Compared with other studies, there is no age limit for completing the questionnaire and participating in the survey.

However, not all students access the “Skuola.net” site to acquire information regarding a given topic; in this study, therefore, even the most deserving students, who prefer to study a given topic on a more reliable source, such as a textbook, could be excluded. Another limitation of the study is not to include, among the questions in the questionnaire, 1 relating to the main source of information regarding the infection caused by SARS-CoV-2: in this way 1 could know those sources least used by students to be able to encourage them to use these sources to increase awareness of the infection. Finally, if the survey had been conducted in the period of maximum contagiousness in Italy, such as the end of March, and in April, the answers of the students could vary, resulting in a greater knowledge of COVID-19, for the several details provided above all by the media, and, probably a more inclined attitude toward the Chinese population.

He and colleagues in their online survey observed social exclusion and discrimination in the outbreak of COVID-19 across the world and inside of China, reporting many feared contact with people from Wuhan or Hubei Province and the stigmatization of people from Hubei was associated with the social exclusion process.^16^

In February 2020, before the first case of COVID-19 was confirmed in Poland, Rzymski and Nowicki conducted an anonymous online survey of Asian medical students in Poland to assess whether they experience any form of prejudice related to the ongoing pandemic. The authors demonstrated the COVID-19 outbreak had triggered xenophobic reactions toward students of Asian-origin before the first SARS-CoV-2 case was confirmed in Poland.^17^

Epidemics spread fear that is the feeling behind the phenomena of racism and xenophobia. The COVID-19 pandemic has uncovered social and political fractures within communities, with racialized and discriminatory responses to fear, disproportionately affecting marginalized groups.^18^

Following the spread of COVID-19 from Wuhan, China, discrimination toward Chinese people has increased. This includes individual acts of microaggression or violence, to collective forms, for example, Chinese people being barred from establishments.^19^

## CONCLUSIONS

Most young people are aware of the main symptoms of the disease and generally show a good level of knowledge about new coronavirus, despite the primary source of information beng social networks, natural docking places of fake news and alarm uncontrolled. Basic notions that, however, do not make them immune to irrational behaviors, from true and proper psychosis. Efforts should be concentrated to increase global awareness of this emerging infection, also to prevent and contrast any prejudicial or discriminatory behaviors, especially among the youngest.

## Supplementary material

For supplementary material accompanying this paper visit http://dx.doi.org/10.1017/dmp.2020.205. click here to view supplementary material

## References

[1] Cui J , Li F , Shi ZL. Origin and evolution of pathogenic coronaviruses. Nat Rev Microbiol. 2019;17(3):181-192. 30531947 10.1038/s41579-018-0118-9 PMC7097006

[2] Weiss SR , Leibowitz JL. Adv Virus Res. 2011;81:85-164. 22094080 10.1016/B978-0-12-385885-6.00009-2 PMC7149603

[3] Su S , Wong G , Shi W , et al. Epidemiology, genetic recombination, and pathogenesis of coronaviruses. Trends Microbiol. 2016;24:490-502. 27012512 10.1016/j.tim.2016.03.003 PMC7125511

[4] Zhu N , Zhang D , Wang WL , et al. A novel coronavirus from patients with pneumonia in China. 2019. N Engl J Med. 2020;382(8):727-733. 31978945 10.1056/NEJMoa2001017 PMC7092803

[5] Huang CL , Wang YM , Li XW , et al. Clinical features of patients infected with 2019 novel coronavirus in Wuhan, China. Lancet. 2020;395(10223):497-506. 31986264 10.1016/S0140-6736(20)30183-5 PMC7159299

[6] World Health Organization. Director-General’s remarks at the media briefing on 2019-nCoV on 11 February 2020 https://www.who.int/dg/speeches/detail/who-director-general-s-remarks-at-the-media-briefing-on-2019-ncov-on-11-february-2020. Accessed February 12, 2020.

[7] Symptoms and diagnosis of Coronavirus infection. Epidemiology for public health, ISS. Epicentro website. https://www.epicentro.iss.it/coronavirus/sintomi-diagnosi Published January 23, 2020. Accessed February 12, 2020.

[8] Bawazir A , Al-Mazroo E , Jradi H , et al. MERS-CoV infection: mind the public knowledge gap. J Infect Public Health. 2018;11(1):89-93. 28647126 10.1016/j.jiph.2017.05.003 PMC7102865

[9] de Michaela L. Coronavirus: la paura del virus e il virus della paura. Chiedilo Qui website. https://www.chiediloqui.it/in-evidenza/il-coronavirus-la-paura-del-virus-e-il-virus-della-paura/ Published February 12, 2020.

[10] STROBE Statement-checklist of items that should be included in reports of case-control studies. Version 4 as published in Oct/Nov 2007 https://www.strobe-statement.org/fileadmin/Strobe/uploads/checklists/STROBE_checklist_v4_case-control.pdf. Accessed July 4, 2020

[11] Elrggal ME , Karami NA , Rafea B , et al. Evaluation of preparedness of healthcare student volunteers against Middle East respiratory syndrome coronavirus (MERS-CoV) in Makkah, Saudi Arabia: a cross-sectional study. Z Gesundh Wiss. 2018;26(6):607-612. 30533343 10.1007/s10389-018-0917-5 PMC6245094

[12] Al-Rabiaahab A , Temsaha MH , Al-Eyadhya AA , et al. Middle East Respiratory Syndrome-Corona Virus (MERS-CoV) associated stress among medical students at a university teaching hospital in Saudi Arabia. J Infect Public Health. 2020;13(5):687-691. 32001194 10.1016/j.jiph.2020.01.005 PMC7102651

[13] Abbag HF , El-Mekki AA , Al Bshabshe AAA , et al. Knowledge and attitude towards the Middle East respiratory syndrome coronavirus among healthcare personnel in the southern region of Saudi Arabia. J Infect Public Health. 2018;11(5):720-722. 29525570 10.1016/j.jiph.2018.02.001 PMC7102736

[14] Nooh HZ , Alshammary RH , Alenezy JM , et al. Public awareness of coronavirus in Al-Jouf region, Saudi Arabia. Z Gesundh Wiss. 2020;1-8. doi: 10.1007/s10389-020-01209-y PMC7088303 32206545

[15] Al-Mohrej A , Agha S. Are Saudi medical students aware of middle east respiratory syndrome coronavirus during an outbreak? J Infect Public Health. 2017;10(4):388-395. 27502524 10.1016/j.jiph.2016.06.013 PMC7102843

[16] He J , He L , Zhou W , et al. Discrimination and social exclusion in the outbreak of COVID-19. Int J Environ Res Public Health. 2020;17(8):2933. 10.3390/ijerph17082933 PMC7215298 32340349

[17] Rzymski P , Nowicki M. COVID-19-related prejudice towards Asian medical students: a consequence of SARS-CoV-2 fears in Poland. J Infect Public Health. 2020;13(6):873-876. doi: 10.1016/j.jiph.2020.04.013 32387102 PMC7196414

[18] Devakumar D , Shannon G , Bhopal SS , et al. Racism and discrimination in COVID-19 responses. Lancet. 2020;395(10231):1194. 10.1016/S0140-6736(20)30792-3 PMC7146645 32246915

[19] Chung RY-N , Li MM. Anti-Chinese sentiment during the 2019-nCoV outbreak. Lancet. 2020;395:686-687. 32122469 10.1016/S0140-6736(20)30358-5 PMC7133618

