# Supplementary material for: How Much Do Young Italians Know About COVID-19 and What Are Their Attitudes Toward SARS-CoV-2? Results of a Cross-Sectional Study
Source: Disaster Med Public Health Prep. 2020 Jun 24:1–7. doi: 10.1017/dmp.2020.205 (PMC7385315; doi:10.1017/dmp.2020.205)
Supplement: Supplementary file 1 [file S1935789320002050sup001.doc]

Table 2a. Univariate analysis (Knowledge questions).

| **Questions** | **Gender** | | **School** | | | | **Macroarea** | | |
| --- | --- | --- | --- | --- | --- | --- | --- | --- | --- |
|  | Male  N(%) | Female  N(%) | University  N (%) | High school  N (%) | Middle school  N (%) | Not attending  N (%) | North  N (%) | Center  N (%) | South  N (%) |
| Q#1: Can Coronavirus infection pass from man to man through cough-borne droplets?  Yes  No  I don’t know | 1609 (76.2)  224 (10.6)  279 (13.2) | 2682 (82.2)  203 (6.2)  377 (11.6) | 796 (83.2)  70 (7.3)  91 (9.5) | 2260 (83.4)  189 (7.0)  261 (9.6) | 505 (77.5)  55 (8.4)  92 (14.1) | 730 (69.2)  113 (10.7)  212 (20.1) | 1979 (82)  166 (6.9)  268 (11.1) | 1026 (77.5)  132 (10.0)  166 (12.5) | 1286 (78.6)  129 (7.9)  222 (13.6) |
| Q#2 Are fever and cough among the signs and symptoms of Coronavirus infection?  Yes  No  I don’t know | 1740 (82.4)  159 (7.5)  213 (10.1) | 2852 (86.6)  112 (3.4)  325 (10) | 862 (90.1)  46 (4.8)  49 (5.1) | 2433 (89.8)  86 (3.2)  191 (7.0) | 517 (79.3)  56 (8.6)  79 (12.1) | 753 (71.4)  83 (7.9)  219 (20.8) | 2119 (87.8)  108 (4.5)  186 (7.7) | 1089 (82.3)  94 (7.1)  141 (10.6) | 1357 (82.9)  69 (4.2)  211 (12.9) |
| Q#3: Is diarrhea among the signs and symptoms of Coronavirus infection?  Yes  No  I don’t know | 236 (11.2)  1327 (62.8)  549 (26) | 340 (10.4)  1952 (59.8)  970 (29.7) | 112 (11.7)  607 (63.4)  238 (24.9) | 272 (10.0)  1744 (64.4)  694 (25.6) | 73 (11.2)  384 (58.9)  195 (29.9) | 119 (11.3)  544 (51.6)  392 (37.2) | 264 (10.9) *  1495 (62) *  654 (27.1) * | 129 (9.7) *  827 (62.5) *  368 (27.8) * | 183 (11.2) *  957 (58.5) *  497 (30.4) * |
| Q#4: How long can infection develop after exposure to Coronavirus?  1d  10-15d  2d  5d  I don’t know | 190 (9.0)  1238 (58.6)  233 (11)  368 (17.4)  83 (3.9) | 277 (8.5)  2133 (65.4)  296 (9.1)  507 (15.5)  49 (1.5) | 57 (6.0)  685 (71.6)  72 (7.5)  132 (13.8)  11 (1.1) | 259 (9.6)  1644 (60.7)  291 (10.7)  484 (17.9)  32 (1.2) | 91 (14.0)  314 (48.2)  101 (15.5)  130 (19.9)  16 (2.5) | 60 (5.7)  728 (69.0)  65 (6.2)  129 (12.2)  73 (6.9) | 202 (8.4)  1526 (63.2)  247 (10.2)  408 (16.9)  30 (1.2) | 112 (8.5)  815 (61.6)  126 (9.5)  208 (15.7)  63 (4.8) | 153 (9.3)  1030 (62.9)  156 (9.5)  259 (15.8)  39 (2.4) |
| Q#5: Can patients with Coronavirus infection be cured?  Yes  No  I don’t know | 1243 (58.9)  457 (21.6)  412 (19.5) | 1963 (60.2)  587 (18.0)  712 (21.8) | 630 (65.8)  170 (17.8)  157 (16.4) | 1590 (58.7)  587 (21.7)  533 (19.7) | 371 (56.9)  137 (21.0)  144 (22.1) | 615 (58.3)  150 (14.2)  290 (27.5) | 1535 (63.6)  459 (19)  419 (17.4) | 745 (56.3)  272 (20.5)  307 (23.2) | 926 (56.6)  313 (19.1)  398 (24.3) |
| Q#6: Is there currently evidence that pet animals. such as dogs or cats. can transmit Coronavirus infection?  Yes  No  I don’t know | 253 (12)  1254 (59.4)  605 (28.6) | 319 (9.8)  1981 (60.7)  962 (29.5) | 82 (8.6)  633 (66.1)  242 (25.3) | 308 (11.4)  1638 (60.4)  764 (28.2) | 111 (17.0)  352 (54.0)  189 (29.0) | 71 (6.7)  612 (58.0)  372 (35.3) | 253 (10.5)  1510 (62.6)  650 (26.9) | 130 (9.8)  788 (59.5)  406 (30.7) | 189 (11.5)  937 (57.2)  511 (31.2) |
| Q#7: Is there currently a vaccine against Coronavirus?  Yes  No  I don’t know | 149 (7.1)  1648 (78)  315 (14.9) | 197 (6.0)  2655 (81.4)  410 (12.6) | 44 (4.6)  823 (86.0)  90 (9.4) | 173 (6.4)  2256 (83.2)  281 (10.4) | 80 (12.3)  483 (74.1)  89 (13.7) | 49 (4.6)  741 (70.2)  265 (25.1) | 150 (6.2)  1992 (82.6)  271 (11.2) | 99 (7.5)  1017 (76.8)  208 (15.7) | 97 (5.9)  1294 (79.0)  246 (15.0) |

All p values are <0.05

*p value is 0.093

Table 2b. Univariate analysis (Attitudes questions).

| **Questions** | **Gender** | | **School** | | | | **Macroarea** | | |
| --- | --- | --- | --- | --- | --- | --- | --- | --- | --- |
|  | Male  N (%) | Female  N (%) | University  N (%) | High school  N (%) | Middle school  N (%) | Not attending  N (%) | North  N (%) | Center  N (%) | South  N (%) |
| Q#9: How has your attitude towards Chinese restaurants changed in this period?  It’s the same  It has improved  It's gotten worse  I don’t know | 1375(65.1)  59 (2.8)  560 (26.5)  118 (5.6) | 2241 (68.7)  131 (4.0)  847 (26.0)  43 (1.3) | 725 (75.8)  17 (1.8)  201 (21.0)  14 (1.5) | 1878 (69.3)  34 (1.3)  769 (28.4)  29 (1.1) | 385 (59.0)  21 (3.2)  228 (35.0)  18 (2.8) | 628 (59.5)  118 (11.2)  209 (19.8)  100 (9.5) | 1717 (71.2)  36 (1.5)  627 (26.0)  33 (1.4) | 842 (63.6)  40 (3.0)  358 (27.0)  84 (6.3) | 1057 (64.6)  114 (7.0)  422 (25.8)  44 (2.7) |
| Q#10: How has your attitude towards Chinese-run shops changed in this period?  It’s the same  It has improved  It's gotten worse  I don’t know | 1405(66.5)  57 (2.7)  532 (25.2)  118 (5.6) | 2349 (72)  127 (3.9)  746 (22.9)  40 (1.2) | 719 (75.1)  12 (1.3)  208 (21.7)  18 (1.9) | 1980 (73.1)  31 (1.1)  672 (24.8)  27 (1.0) | 402 (61.7)  19 (2.9)  213 (32.7)  18 (2.8) | 653 (61.9)  122 (11.6)  185 (17.5)  95 (9.0) | 1795 (74.4)  35 (1.5)  546 (22.6)  37 (1.5) | 895 (67.6)  41 (3.1)  307 (23.2)  81 (6.1) | 1064 (65.0)  108 (6.6)  425 (26.0)  40 (2.4) |
| Q#11: How has your attitude towards Chinese tourists changed in this period?  It’s the same  It has improved  It's gotten worse  I don’t know | 118 (52.9)  48 (2.3)  828 (39.2)  118 (5.6) | 1988 (60.9)  129 (4.0)  1108 (34.0)  37 (1.1) | 629 (65.7)  15 (1.6)  297 (31.0)  16 (1.7) | 1572 (58.0)  33 (1.2)  1079 (39.8)  26 (1.0) | 322 (49.4)  14 (2.1)  298 (45.7)  18 (2.8) | 583 (55.3)  115 (10.9)  262 (24.8)  95 (9.0) | 1455 (60.3)  37 (1.5)  887 (36.8)  34 (1.4) | 714 (53.9)  33 (2.5)  499 (37.7)  78 (5.9) | 937 (57.2)  107 (6.5)  550 (33.6)  43 (2.6) |
| Q#12: If in the class you attend there was a student of Chinese origins how would you behave?  It’s the same  I would avoid contacts  I would urge them not to come  I would put on the mask  I don’t know | 1526(72.3)  262 (12.4)  98 (4.6)  94 (4.5)  132 (6.3) | 2641 (81.0)  302 (9.3)  178 (5.5)  116 (3.6)  25 (0.8) | 790 (64.2)  89 (9.3)  29 (3.0)  28 (2.9)  21 (2.2) | 2256 (83.2)  269 (9.9)  67 (2.5)  95 (3.5)  23 (0.8) | 444 (68.1)  110 (16.9)  37 (5.7)  49 (7.5)  12 (1.8) | 677 (64.2)  96 (9.1)  143 (13.6)  38 (3.6)  101 (9.6) | 1961 (81.3)  258 (10.7)  65 (2.7)  97 (4.0)  32 (1.3) | 989 (74.7)  142 (10.7)  59 (4.5)  34 (2.6)  100 (7.6) | 1217 (74.3)  164 (10.0)  152 (9.3)  79 (4.8)  25 (1.5) |

All p values are <0.05
